# Electromagnetic Interference in Patients with Implanted Cardioverter-Defibrillators and Implantable Loop Recorders

**Published:** 2002-07-01

**Authors:** Marcos de Sousa, Gunnar Klein, Thomas Korte, Michael Niehaus

**Affiliations:** Department of Cardiology and Angiology, Medical School Hannover, Germany

## Introduction

Modern life exposes us all to an ever-increasing number of potential sources of electromagnetic interference (EMI) and patients with Implantable rhythm devices (IRD) like pacemakers, implantable cardioverter defibrillators or implantable loop recorders often ask about the use of microwave ovens, walking through airport metal detectors and the use of cellular phones.

Electromagnetic interference occurs when electromagnetic waves emitted by one device impede the normal function of another electronic device. The potential for interaction between implanted pacing systems and cardioverter-defibrillators (electromagnetic interference, EMI) has been recognized for years [1-4]. It has been shown that EMI can produce clinically significant effects on patients with implanted pacemakers and ICDs. For these reasons the following text discusses the influence of several EMI generating devices on IRD.

## Mobile phones

These phones have the potential to interfere with pacemaker- or ICD function. Several studies have shown that cellular phones might cause electromagnetic interference with complex medical equipment [5,6] including pacemakers [7-11]. The effects of digital cellular telephones on patients with ICDs have been studied in a relatively small number of patients with various ICD models provided by a single manufacturer. The static magnetic field generated by the cellular telephone when placed at very close proximity to the ICD during in vitro testing has caused temporary suspension of ventricular tachycardia and ventricular fibrillation detection.

European cellular phones are different from those used in North America: The NADC-phones (North American Digital Cellular) work on a carrier frequency of 835 MHz. For data transmission a pulse amplitude modulation of 50 pulses/s is used (TDMA-50) and the peak power of the handset is limited to 0.6W. In contrast, the peak power of digital phones used in the European GSM-net is 2W for D-net and 1W for E-net [12,13]. The D-net works on a carrier frequency of 900 MHz modulated with 217 Hz; the E-net works on a carrier frequency of 1800 MHz.

How the North American cellular phones are different to those used in Europe the susceptibility of tiered single chamber ICDs to EMI or other dysfunction caused by commercially available digital mobile telephones was evaluated in our own study. For our evaluations, two different types of European digital cellular phone systems were used.

We prospectively analyzed 97 patients with different ICDs and exposed them to two different types of European digital cellular handy phones (Ericsson GH337, 900 MHz and NOKIA NHK1EA, 1,8GHz). The effect of high radiofrequency-output (RF) was tested during continuous recording of the marker channel and intracardiac ECG. During the recordings the handsets were put in a calling position close to the patient's ear and on top of the device. We noticed interferences (loss of communication or temporary inactivation of the device during interrogation) in 38 patients; most of them (93%) during testing close to the device.

Main finding of our study was that electromagnetic interference transmitted by digital cellular handheld D- or E-net phones, commonly used in Europe did not interfere with normal ICD-function of tested single-chamber devices under daily-life conditions. Inappropriate sensing and detection of ventricular tachyarrhythmias were not found. These observations are in accordance with the results described by Fetter et al [17], Occhetta [14], Barbaro et al [15] and Jimenez et al[16]. In contrast to Fetter et al. we did not see any temporary suspension of the ICD function by static magnetic field (magnetic reversion counter=0) generated by the speaker in the cellular phone's earpiece which may be in part explained by a less strong static magnetic field of the evaluated GSM-phones. Implantation technique did not have any relation to interference with the function of the ICD.

We concluded that there is no evidence of harm related to the use of the tested European GSM handsets for the tested single chamber ICDs independent of the used GSM-net. Since most interferences were seen when the GSM-phones were in a short distance to the ICDs, patients should be advised not to carry their GSM-phone close to the device. This is in accordance to in vitro studies with American GSM-phones[18].

As ICD-interrogation is the most susceptible phase for interference, the use of GSM-phones should be prohibited in hospital areas where interrogation takes place.

For implantable loop recorders there are only small experiences so far[17]. It could be shown that GSM phones did not affect appropriate function of the implantable loop recorder.

## Microwave ovens

Although no recent studies have been performed which test the effect of household microwave energy on pacemakers and ICDs, it is widely believed and accepted that all modern pacemakers are adequately shielded from microwave energy produced by modern appliances[18]. Pacemaker manufacturers therefore recommend that patients with implanted devices do not need to take special precautions in the use of microwave ovens or other common household equipment such as televisions, radios, toasters and electric blankets.

## Metal-detector gates

The effect of metal-detector gates on implanted pacemakers has been studied more than 10 years ago[19]. In 103 patients who were monitored as they passed through typical metal detectors alarms invariably were activated when the patients walked through the gates. In none of the patients the pacemaker function was affected: None of the devices was reset to the programmed noise-protection mode (most often VOO) or spontaneous fixed rate mode of function, nor were any of the devices' outputs inhibited in paced patients, or inappropriately delivered in patients who had normal cardiac rhythm. Test series with ILRs did not show any interference with metal detector gates. It is therefore accepted practice to advise the patient that while airport screening devices may detect the pacemaker, ICD or ILR metal case the device will not be adversely affected. Patients should carry their device identification card for the purpose of obtaining security clearance.

## Electronic article surveillance systems

 Electronic article surveillance systems have recently been recognized as having the potential to interact with implanted rhythm devices[20]. The commercial use of such scanning devices is widespread, and case reports have been published in which patients received inappropriate ICD therapies while lingering between or touching electronic article surveillance gates. Because a very large number of electronic article surveillance devices are in use, and because only very few episodes of possible interaction between electronic article surveillance devices and pacemakers or ICDs have been reported, it is likely that too much is being made of this issue.

Electronic surveillance systems use three different technologies to detect the presence of a metal alloy tag within an electromagnetic field created between two parallel gates: magnetic audio frequency, swept frequency, and acoustomagnetic or pulsed low-frequency. The detection of such a tag signals a theft. The literature suggests, that significant EMI with implanted rhythm devices is most likely to occur with the acoustomagnetic mode of electronic article surveillance, and that pacemakers are more likely to be affected than ICDs, likely due to electromagnetic fields of six different electronic article surveillance devices, no instance of significant interference with normal ICD function was seen. The testing protocol included positioning the patients for five minutes between the electronic article surveillance gates while rotating 360° as well as leaning against the electronic article surveillance transmitter. In another study, patients with ICDs with pacing capability performed routine walking through electronic article surveillance gates as well as prolonged exposure within the gates, with and without pacing from the implanted device. The absence of significant interaction between electronic article surveillance gates and ICDs was confirmed during walking at a normal slow pace through the gates. Under conditions of extreme exposure, however, seven of 169 patients exhibited some interaction between the ICD and the electronic article surveillance device, manifested by noise-sensing that resulted in complete or prolonged inhibition of the device output. Such output inhibition might have been clinically relevant, and could also have resulted in inappropriate ICD shocks had this function not been suspended during the testing protocol. Older-generation devices, and those implanted in the abdomen were more likely than newer-generation subpectorally implanted ones to manifest these interactions. In general, electronic surveillance systems do not pose a threat to the tachycardia functions of ICDs under reasonably normal conditions. More prolonged exposures or closer proximity to the transmitter can result in inappropriate shocks.

In contrast to ICDs, acoustomagnetic electronic surveillance devices can interact with permanent pacemakers. Asynchronous pacing (noise-reversion), atrial and ventricular oversensing and surveillance device-induced pacing have been described during a real-life walk through the gates. No pacemaker was reprogrammed, and no patient experienced severe symptoms. No difference in electronic article surveillance device effect on pacemakers was observed between unipolar and bipolar sensing configurations. Since these effects on pacemakers occur only while the patient is within the electronic article surveillance device's magnetic field, it is prudent to advise patients to avoid prolonged exposure to electronic article surveillance systems, lingering within the surveillance gates and direct contact with the gates.

Acoustomagnetic electronic surveillance devices can interact with permanent pacemakers. Asynchronous pacing (noise-reversion), atrial and ventricular oversensing and surveillance device-induced pacing have been described during a real-life walk through the gates. No difference in electronic article surveillance device effect on pacemakers was observed between unipolar and bipolar sensing configurations. Since these effects on pacemakers occur only while the patient is within the electronic article surveillance device's magnetic field, it is prudent to advise patients to avoid prolonged exposure to electronic article surveillance systems and direct contact with the gates. Although data are lacking, it is likely that the same considerations and cautions apply to the pacing functions of ICDs which are part of all currently available systems.

For ILR systems it has been shown recently that their function can be impaired by electronic surveillance systems.

## Magnetic resonance imaging

The safety of magnetic resonance imaging in patients with IRD has been debated for years. In general, the presence of these devices is an absolute contraindication to the performance of magnetic resonance imaging, since cardiac pacing and total inhibition of output can occur during magnetic resonance exposure[21]. However, it has been suggested that if patients are positioned so that the thorax does not enter the magnet bore no significant interaction occurs. Furthermore, it has been shown[22] that MR imaging at 0.5 T can be safely performed in patients with implanted pacemakers in carefully selected clinical circumstances when appropriate strategies (programming to an asynchronous mode, adequate monitoring techniques, limited RF exposure) are used.

These data need to be confirmed before magnetic resonance imaging of the extremities can be allowed in patients with implanted devices.

First test series with ILRs have resulted in an irreversible error in one nonimplanted device[18]. Therefore MRI diagnostics should be scheduled before implantation of an ILR system.

## Electrocautery devices

Electrocautery devices have long been known to have the potential for interfering with pacemaker function. These devices generate a high energy electromagnetic field with a frequency that may pass through the filters of ICDs. This may result in oversensing, independent of unipolar or bipolar coagulation mode is used. This oversensing leads to a pacemaker inhibition or false detection of ventricular tachyarrhythmias. Therefore, for surgical procedures using electrocautery devices, pacemaker-dependent patients should be programmed into an asynchronous pacing mode. Usually, ICD-patients should be programmed to detection-off using a programmer. Nevertheless, detection of ICDs may be temporarily inactivated using a pacemaker magnet placed above the device on condition that monitoring and an external defibrillator are available.

## Radiotherapy

The CMOS electronic circuitry of pacemaker, ICD and ILR-devices which is currently used in all pacemakers and ICDs is responsible for the high sensitivity of these devices to ionizing radiation. Nevertheless, no large recent studies have been performed which test the effect of radiotherapy on pacemakers and ICDs. 1991 Rodriguez et al[23] could show severe malfunctions of pacemakers and ICDs: Of the 17 pacemakers exposed to photon radiation eight failed before 50 Gy, whereas four of the six pacemakers exposed to electron radiation failed before 70 Gy. For the ICDs, detection and charging time increased with accumulated radiation dose and charging time increased catastrophically at less than 50 total pulses delivered when compared with the charging time of six ICDs implanted at the same time. In 1995 Roethig et al[24] showed similar results using 9-MV photon radiation. Our own experience in 3 ICD-patients who underwent radiation therapy with a cumulative dose of <5 Gy showed no damage of the device, oversensing or inhibition of pacing.

Therefore, direct radiation of pacemakers or ICDs at therapeutic levels should be strictly avoided. Furthermore, pacemaker and ICDs have to be controlled in short periods during and after radiation therapy, and pacemaker or ICDs should be exchanged after the radiotherapy when accumulative dose on the pacemaker exceeds 5 Gy.
